# The incidence of severe oral mucositis in patients undergoing different conditioning regimens in haematopoietic stem cell transplantation

**DOI:** 10.1007/s00520-022-07328-4

**Published:** 2022-08-26

**Authors:** Midori Nakagaki, Glen A. Kennedy, Nicole C. Gavin, Alexandra Clavarino, Karen Whitfield

**Affiliations:** 1 Pharmacy Department, Royal Brisbane and Women’s Hospital, Brisbane, Australia; 2 School of Pharmacy, The University of Queensland, Brisbane, Australia; 3 Cancer Care Services, Royal Brisbane and Women’s Hospital, Brisbane, Australia; 4 School of Medicine, The University of Queensland, Brisbane, Australia; 5 School of Nursing, Midwifery and Social Work, The University of Queensland, Brisbane, Australia; 6 School of Nursing, Queensland University of Technology, Brisbane, Australia; 7 Faculty of Public Health, The University of Queensland, Brisbane, Australia

**Keywords:** Oral mucositis, Haematopoietic stem cell transplantation, Conditioning, Risk factors

## Abstract

**Purpose:**

Oral mucositis is a common complication during haematopoietic stem cell transplantation (HSCT). This study aimed to assess the incidence of severe mucositis in patients undergoing different HSCT regimens.

**Methods:**

This single-centre retrospective study reviewed daily oral assessment for 467 consecutive patients who underwent different transplant regimens for matched unrelated or related allogeneic HSCT with post-transplant methotrexate, haploidentical or mismatched HSCT with post-transplant cyclophosphamide (PTCy), or autologous HSCT. Oral care and cryotherapy with melphalan were used. Patient demographic data, oral mucositis WHO grade, use of total parenteral nutrition (TPN) and patient-controlled analgesia (PCA) were collected.

**Results:**

Grade 3–4 oral mucositis was common in myeloablative total body irradiation (TBI)-based regimens cyclophosphamide/ TBI (CyTBI) (71%) and fludarabine/ TBI (FluTBI) with PTCy (46%), as well as reduced-intensity fludarabine/melphalan (FluMel) (43%) and carmustine/etoposide/cytarabine/melphalan (BEAM) autologous HSCT (41%). In contrast, grade 3–4 oral mucositis was less common in reduced-intensity haploidentical regimen melphalan/fludarabine/TBI with PTCy (19%), all non-myeloablative regimens (0–9%) and high-dose melphalan autologous HSCT (26%). TPN and PCA use were correlated to oral mucositis severity.

**Conclusions:**

Severe oral mucositis was associated with myeloablative TBI, methotrexate and melphalan in combination with methotrexate and in BEAM. Use of PTCy was preferable over methotrexate to prevent oral mucositis.

## Introduction

Oral mucositis (OM) is a common complication during haematopoietic stem cell transplantation (HSCT). OM is experienced by 70–86.8% of patients undergoing HSCT [1] and reported as the single most debilitating side effect [2].

For the prevention of OM, the Multinational Association of Supportive Care in Cancer/International Society of Oral Oncology (MASCC/ISOO) guidelines recommend oral care, cryotherapy during melphalan administration, palifermin for total body irradiation (TBI)-based autologous HSCT, and photobiomodulation (previously known as low-level laser therapy) [3]. Several other interventions have been tested, including antimicrobials, vitamins and minerals. However, there is no high-quality evidence to support these inexpensive and simple interventions.

Undertaking OM studies in HSCT is challenging because of the heterogeneity of the patients. There are many confounding factors, both patient-related (e.g. age, gender, disease, body size) and treatment-related (e.g. type of HSCT, conditioning regimens). Reported patient-related risk factors for OM include female gender [4–6], age (conflicting reports) [7, 8] and lower body mass index (BMI) [6, 8]. In recent years, oral microbiome and genetic factors have been suggested as risk determinants for OM [9, 10]. Treatment-related risk factors have been more frequently analysed in the literature. Impact of conditioning chemotherapy intensity has conflicting data, and it is not clear whether intensity per se is an independent risk factor [8, 11–14]. Methotrexate (MTX) as an immunosuppressant, and its dose, are consistently reported as risk factors for OM [8, 13, 15]. Melphalan is known to cause OM and its dose and administration method appear to affect OM [16, 17]. A large study reported that high-dose melphalan containing regimens caused the most severe OM [18]. Only a small number of OM studies control for these confounding factors, which may affect OM more than the interventions tested. When interventions are tested in clinical trials, it is essential to stratify randomisation according to the most important confounding factor and monitor as many confounding factors as possible.

This study aimed to retrospectively review the incidence of severe OM in patients undergoing different conditioning regimens used in matched allogeneic, haploidentical, and autologous HSCT before a planned randomised study to determine the randomisation strategies. In addition, other risk factors were reviewed in patients who had received the same regimen.

## Patients and methods

### Study design

This single-centre, retrospective study reviewed the incidence of severe OM in patients undergoing different HSCT regimens. Patients’ gender, age, and BMI were evaluated as potential risk factors in patients receiving the same regimen. This study was approved by the institutional Human Research Ethics Committee (HREC/2020/QRBW/60530).

### Patients

**Fig. 1:**
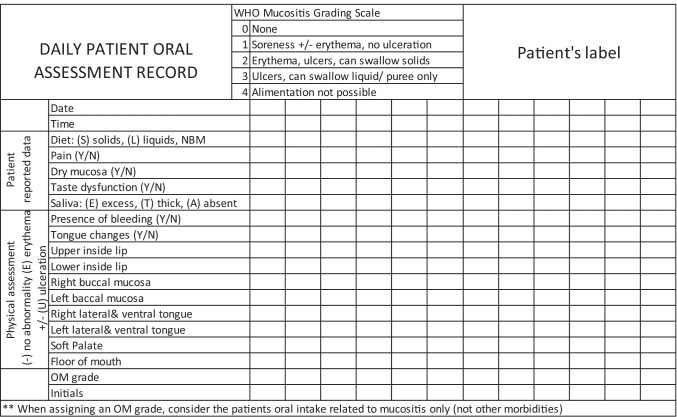
Daily oral assessment sheet routinely used in the HSCT unit

This study was conducted in an Australian tertiary adult hospital. In the hospital, approximately 150 HSCT are performed per year, of which approximately 75% are allogeneic. Patients who underwent allogeneic or autologous HSCT from January 2017 to June 2020 were included. Most patients received HSCT as hospital inpatients at least until neutrophil recovery. All patients on the HSCT ward had daily oral assessments according to the unit practice, which were available in the electronic medical record (Fig. 1). Patients were excluded when their oral assessment data was not available due to early transfer to other hospitals or the Intensive Care Unit where oral assessment is not conducted, or for any other reasons.

### Treatment

Patients underwent different transplant regimens for matched unrelated or related allogeneic HSCT, haploidentical or mismatched HSCT with post-transplant cyclophosphamide (PTCy), or autologous HSCT. All patients received supportive medications according to the unit guidelines, including immunosuppressants, antibiotic, antiviral, antifungal, *Pneumocystis jirovecii pheumonia* prophylaxis, proton pump inhibitors, standard antiemetics and vitamin/electrolyte supplements. With matched allogeneic HSCT, the immunosuppressants were cyclosporin from day − 1 and MTX at 15 mg/m^2^ on day + 1 and 10 mg/m^2^ on days + 3, + 6 and + 11, with the exception of fludarabine/low-dose TBI (FluTBI mini), where mycophenolate was used instead of MTX. With haploidentical or mismatched HSCT, the immunosuppressants were PTCy 50 mg/kg on days + 3 and + 4, and tacrolimus and mycophenolate from day + 5. For OM prevention, patients received saline and sodium bicarbonate mouthwashes four times a day from hospital admission until discharge. If patients received high-dose melphalan-containing regimens, cryotherapy was provided from 10 min before until 2 h after melphalan infusion. Patients were allowed to use their favourite frozen products if they wished. If patients developed severe mucositis pain that was not controlled with pro re nata opioid administration, patient-controlled analgesia (PCA) was provided. If patients’ oral intake decreased below 50% of daily requirements, total parenteral nutrition (TPN) was commenced. As a unit policy, TPN use was generally avoided in autologous HSCT patients, due to expected limited duration of need. From July 2019, the HSCT unit introduced a routine use of enteral feeding for all allogeneic HSCT patients and as such, after this time TPN use was limited to patients not tolerating nasogastric tube insertion or enteral feeds.

### OM assessment

As part of routine patient care, nurses assessed patients’ OM daily from hospital admission until OM healing or neutrophil recovery, whichever was the later (Fig. 1). Nursing staff asked patients and recorded oral intake, oral pain (yes/no), dry mucosa (yes/no), taste dysfunction (yes/no), and amount of saliva (excess/normal/thick/absent). Nurses assessed patients’ oral cavity and recorded the presence of erythema and ulcers in eight different parts of the mouth. Finally, nursing staff graded OM according to the World Health Organisation (WHO) oral mucositis scale with clarifications as per European audit [19, 20]. The WHO grades were defined as follows: grade 0: none or erythema (no pain), grade 1: soreness ± erythema, no ulcers, grade 2: ulcers, able to eat a solid diet, grade 3: ulcers, tolerates liquid only, grade 4: ulcers, not able to tolerate a solid or liquid [20]. All new nurses to the unit are trained to perform oral assessment by senior nurses in the unit. If the WHO grade did not match the documented nursing observation (oral intake, pain and ulcer), the grade was corrected by the research team.

### Other data collection

Patient demographics (gender, age, height, weight and diagnosis), the use of TPN and PCA were collected. After June 2019 when routine enteral feeding was introduced, patients who received enteral feeds alone (*N* = 80) were excluded from data analysis for TPN use. All data was captured from initial admission for HSCT until recovery from transplant and did not include OM or TPN use due to GVHD.

### Study endpoints

The primary endpoint was the incidence of grade 3 to 4 OM. Secondary endpoints were the incidence of grade 2 to 4 OM, duration of grade 3 to 4 and 2 to 4 OM, TPN and PCA use. These endpoints were compared between different conditioning regimens. In patients who underwent allogeneic fludarabine/melphalan (FluMel) conditioning, these endpoints were compared between genders. In patients who received FluMel, the relationships between OM severity and patients’ age/BMI were also evaluated.

### Data analysis

The incidence of grade 3 to 4 and grade 2 to 4 OM, TPN and PCA use in patients that underwent different regimens were analysed using descriptive statistics. In FluMel patients, the differences between gender were evaluated using the Fisher’s exact test. The influences of BMI and age were evaluated using the student *t*-test.

## Results

### Patients

From January 2017 to June 2020, 515 patients received allogeneic or autologous HSCT. In total, 48 patients were excluded due to lack of documented OM grades in the patients’ medical records (transfer to other hospital: *N* = 32, early discharge: *N* = 4, intensive care unit admission: *N* = 4, early death: *N* = 1, other reasons: *N* = 7). A total of 467 patients were evaluated. Median age was 56 (range 18–76) and 40% (*N* = 186) were female.

### Regimens and the incidence of grade 3–4 OM (primary endpoint)

Table 1 shows the intensity of regimens, numbers of patients, total doses of chemotherapy and radiation in each regimen and the incidence of grade 3 to 4 OM. Conditioning schedules are also shown at the bottom. Grade 3 to 4 oral mucositis was common in myeloablative TBI-based regimens (CyTBI and FluTBI with PTCy) as well as reduced intensity matched allogeneic protocols (FluMel) and BEAM autologous HSCT. Grade 3 to 4 oral mucositis was less commonly experienced in reduced intensity haploidentical regimens (MelFluTBI with PTCy), all non-myeloablative regimens (FluCy, FluTBI mini and FluCyTBI with PTCy) and high-dose melphalan (HDM) autologous HSCT.

**Table 1:** HSCT regimens and G3 to 4 OM

Regimens	Intensity	*N*	TBI (Gy)	Mel (mg/m^2^)	Flu (mg/m^2^)	Cy (mg/m^2^)	PTCy (mg/kg)	MTX (mg/m^2^)	G3–4 OM (%)
CyTBI	MAC	76	12			120		45	71
FluTBI (PTCy)	MAC	13	12		90		100		46
FluMel	RIC	197		120	125			45	43
BEAM	Auto	34		140	(with carmustine, etoposide, cytarabine)				41
HDM	Auto	76		200					26
MelFluTBI (PTCy)	RIC	27	2	100	160		100		19
FluCy	NMC	11			125	120		45	9
FluCyTBI (PTCy)	NMC	7	2		150	29	100		0
FluTBI mini	NMC	7	2		90				0
Other regimens		19							

*PTCy*, post-transplant cyclophosphamide; *MAC*, myeloablative; *RIC*, reduced intensity; *NM*, non-myeloablative; *TBI*, total body irradiation; *Mel*, melphalan; *Flu*, fludarabine; *Cy*, cyclophosphamide; *MTX*, methotrexate; *G3-4 OM*, grade 3 to 4 oral mucositis

Conditioning schedules (pre-transplant)

CyTBI: Cy 60 mg/kg days − 5, − 4, TBI 2 Gy BD on days − 3, − 2, − 1

FluTBI (PTCy): flu 30 mg/m^2^ days − 7 to − 5, TBI 1.5 Gy BD days − 4 to − 1

FluMel: flu 25 mg/m^2^ days − 7 to − 3, Mel 120 mg/m^2^ on day − 2

BEAM: carmustine 300 mg/m^2^ day − 6, cytarabine 200 mg/m^2^ BD days − 5 to − 2, etoposide 200 mg/m^2^ days − 5 to − 2, Mel 140 mg/m^2^ day − 1

HDM: Mel 200 mg/m^2^ day − 1

MelFluTBI (PTCy): Mel 100 mg/m^2^ day − 6, flu 40 mg/m^2^ days − 5 to − 2, TBI 2 Gy day − 1

FluCy: flu 25 mg/m^2^ days − 8 to − 4, Cy 60 mg/kg days − 3, − 2

FluCyTBI: flu 30 mg/m^2^ days − 6 to − 2, Cy 14.5 mg/kg days − 6, − 5, TBI 2 Gy day − 1

FluTBI mini: flu 30 mg/m^2^ days − 4 to − 2, TBI 2 Gy day − 1

### Secondary endpoints

**Fig. 2:**
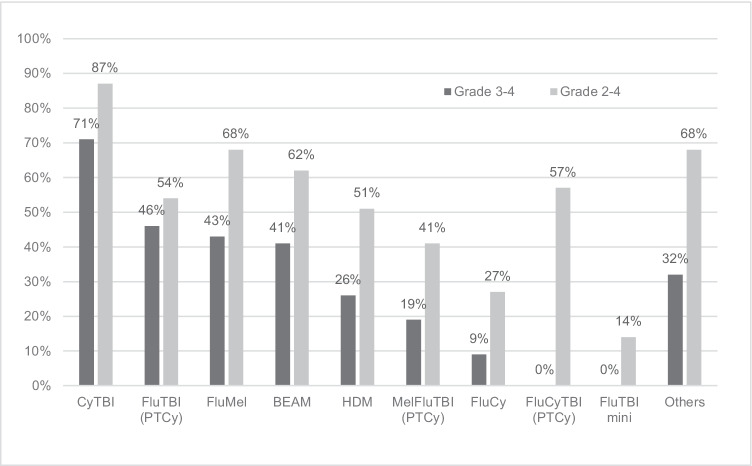
Incidence of grade 3–4 and grade 2–4 oral mucositis. Refer Table 1 for details of conditioning regimens

**Fig. 3:**
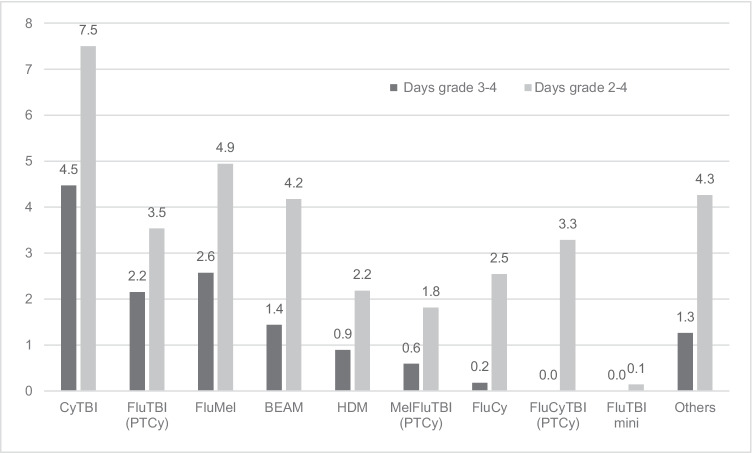
Mean duration (days) of grade 3 to 4 and grade 2 to 4 oral mucositis. Refer Table 1 for details of conditioning regimens

Figures 2 and 3 demonstrate the incidence and mean days of grade 3 to 4 and grade 2 to 4 OM. Similar trends were observed with the primary endpoint. Grade 2 to 4 OM, durations of grade 3 to 4 and grade 2 to 4 OM were higher in CyTBI, followed by FluMel and BEAM. Myeloablative FluTBI had a lower incidence of grade 2 to 4 OM compared to FluMel and BEAM.

**Fig. 4:**
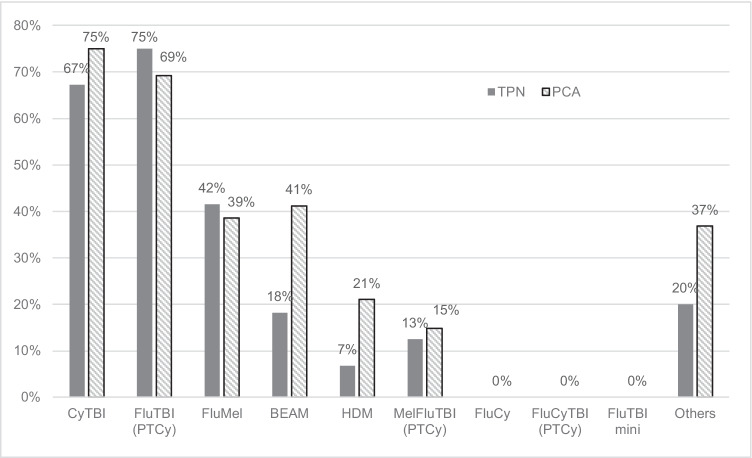
Use of TPN and PCA. TPN, total parenteral nutrition; PCA, patient-controlled analgesia. Refer Table 1 for details of conditioning regimens

Figure 4 shows the use of TPN and PCA in patients receiving different regimens. Similar to the incidence of grade 3 to 4 OM, PCA was most commonly used in myeloablative TBI-based regimens, followed by FluMel and BEAM. TPN use was similar, except lower use in autologous regimens, as expected by unit guidelines.

### Patient-related risk factors in FluMel patients

#### The incidence of grade 3 to 4 and grade 2 to 4 OM

**Fig. 5:**
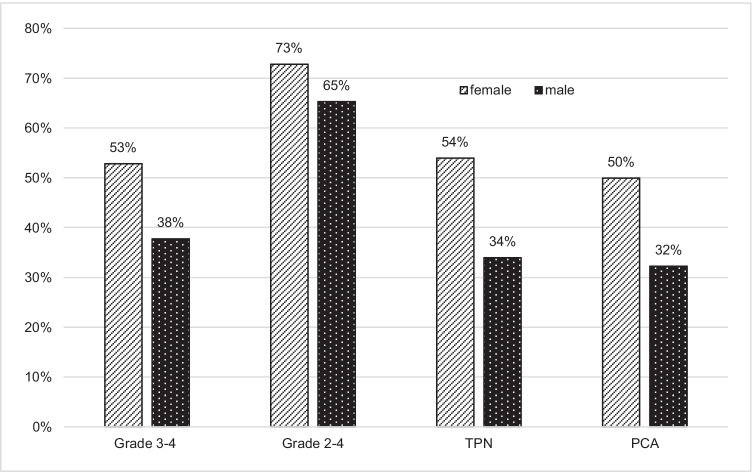
Incidence of oral mucositis and TPN/PCA use in gender received FluMel (*N* = 197, female = 70, male = 127). The incidence of grade 3 to 4 OM (*p* = 0.05), TPN use (*p* = 0.02) and PCA use (*p* = 0.02) were significantly higher in female. The incidence of grade 2 to 4 OM (*p* = 0.34) was nonsignificantly higher in female patients. TPN, total parenteral nutrition; PCA, patient-controlled analgesia; FluMel, fludarabine/melphalan; OM, oral mucositis

Use of TPN and PCA were compared between male and female patients who underwent FluMel conditioning (Fig. 5). The incidence of grade 3 to 4 OM (53 vs. 38%, *p* = 0.05), TPN use (54 vs. 34%, *p* = 0.02) and PCA use (50 vs. 32%, *p* = 0.02) were significantly higher in female patients compared to male patients. The incidence of grade 2 to 4 OM (73 vs. 65%, *p* = 0.34) was not significantly higher in female patients. The median BMIs in patients who developed grade 0–4 OM were 27 (grade 0), 26 (grade 1), 27 (grade 2), 26 (grade 3) and 28 (grade 4) respectively. The median ages were 59 (grade 0), 61 (grade 1), 59 (grade 2), 60 (grade 3) and 60 (grade 4). The BMI and age did not vary across the patients who developed different grade OM.

## Discussion

The incidence of grade 3 to 4 OM differed depending on the conditioning regimens. A lower incidence was observed with FluTBI mini, FluCyTBI (PTCy), FluCy and MelFluTBI (PTCy). This indicates that 2 Gy TBI, fludarabine and high-dose cyclophosphamide are unlikely to cause severe OM. Therefore, the higher incidence of grade 3 to 4 OM in CyTBI and FluTBI is mostly due to myeloablative high-dose TBI and this is considered as radiation-induced OM. When comparing similar regimens, CyTBI vs. FluTBI (PTCy), FluMel vs. MelFluTBI (PTCy) and FluCy vs. FluCyTBI (PTCy), the use of MTX as an immunosuppressant appears to be a risk factor compared to PTCy. Melphalan is known to cause OM. However, given the relatively low incidence of grade 3 to 4 OM in patients who received HDM autologous and Mel FluTBI (PTCy), it appears that melphalan is a risk factor when combined with MTX or in BEAM rather than the dose of melphalan itself. Among the commonly used regimens, the incidence of grade 3 to 4 OM is highest in CyTBI, then FluMel and BEAM followed by HDM.

A higher incidence of grade 3 to 4 OM has been previously observed with myeloablative conditioning compared to reduced-intensity conditioning utilizing busulfan-based regimens [12]. The authors reported grade 3 to 4 OM in 45% of patients receiving a myeloablative regimen, which is lower than grade 3 to 4 OM experienced in our CyTBI cohort (71%) and comparable to our FluTBI and FluMel cohorts (46% and 43%). A systematic review that included various regimens did not identify any difference in incidence and severity of OM between myeloablative and reduced-intensity conditionings [13]. The risk of OM appears to be determined by specific drug combinations and use of MTX-based immunosuppression (in allogeneic HSCT) rather than the conditioning intensity alone. Methotrexate, as an immunosuppressant, has previously been reported as a risk factor for OM [8, 13, 15]. Our study corroborates this, and suggested superiority of PTCy in terms of OM prevention.

Our results are different from earlier research by Wardley et al. [18] and Blijlevens et al. [21]. Their research identified high-dose melphalan as a major regimen-related risk factor. These studies were conducted before the routine use of cryotherapy with melphalan. Our study used routine 2-hour cryotherapy with melphalan, and even the highest 200 mg/m^2^ HDM regimen was associated with relatively low grade 3 to 4 incidence (28%). The difference between our study and older studies were most likely due to cryotherapy with melphalan. Although melphalan dose has been shown to predict OM in one study that did not mention cryotherapy [16], in our experience, melphalan doses were not relevant to the incidence of grade 3 to 4 or grade 2 to 4 OM. Use of melphalan in combination with other agents in conditioning (carmustine and etoposide) or GVHD prevention (MTX) appeared to be needed for development of OM. As cryotherapy lowered the risk of melphalan, the risk of other chemotherapy, TBI and MTX may have stood out. Cryotherapy with melphalan is recommended in the current MASCC/ISOO guidelines [3]. It should be standard of care due to evidence, low cost, accessibility and no requirement for staff training. When cryotherapy is routinely used, high-dose melphalan may no longer be a very high-risk drug unless it is combined with other high-risk drugs.

The secondary outcomes showed similar trends with the incidence on grade 3 to 4 OM. The incidence and duration of grade 2 to 4 OM were relatively lower in FluTBI (PTCy) and higher in FluCyTBI (PTCy) compared to the primary outcome. Small sample size in these regimens may have affected the results. PCA use was correlated to the incidence of grade 3 to 4 OM. The use of TPN was lower in BEAM and HDM autologous patients, which reflected the unit’s practice of limiting TPN in autologous patients.

There are other reported risk factors than HSCT regimens, including younger age, female gender and lower BMI. However, it is difficult to assess these factors independently. For example, younger patients may have higher risks because they are more likely to have myeloablative regimens. In our study, 197 (42%) patients underwent FluMel allogeneic HSCT. In this population, the incidence of grade 3 to 4 OM, use of TPN and PCA were higher in female patients. As all patients underwent the same treatment, the effect of treatment-related risk factors was minimised. Female gender has been consistently reported as a patient-related risk factor [4–6]. This could be because females tend to be smaller and if chemotherapy is calculated with body surface area, they generally receive a higher dose per kg. Another explanation may be the negative effects of female sex hormones as pointed out in a recent study [4]. The gender difference in the incidence of grade 2 to 4 OM was not statistically significant. Grade 2 to 4 OM is determined by the presence of ulcers and more objective than other measures. Therefore, the gender differences in grade 3–4 OM, TPN and PCA uses may be interpreted partly as gender difference of pain perception and oral intake affected by pain. A large study demonstrated that women report increased clinical pain compared to men [22].

In our study, median BMI and age did not vary across the patients with different OM grades after FluMel conditioning. This finding is at variance with the results of a study published by Shouval et al. in 2019 [8]. This study involved a heterogeneous patient cohort receiving a wide range of conditioning regimens, and their multivariate analysis did not show age and BMI as significant risk factors.

In our study, it was confirmed that severe OM is associated with the HSCT conditioning and immunosuppressant regimens containing myeloablative TBI, methotrexate and melphalan in combination with methotrexate or in BEAM. Currently, the only FDA-approved prophylaxis of OM is palifermin. Palifermin appears to be effective only for TBI-induced OM and there are associated costs and accessibility issues with this medication. Clearly, studies to identify more accessible and inexpensive interventions are required. Challenges in OM studies include heterogenicity of patients, disease, and treatment. Based on our study and other published studies, when conducting a clinical trial, it is appropriate to stratify randomisation according to the conditioning and immunosuppressant regimens if the study includes patients undergoing different regimens.

Our study had some limitations. First of all, this was a retrospective study using daily OM assessment by nurses. In a busy HSCT unit, we observed their OM gradings were not always accurate [23]. While all WHO grades were reviewed against recorded observations, due to the retrospective nature of the study, we had to rely on their observations. Secondly, HSCT regimens were dramatically changed towards the end of study period from matched unrelated donor allograft regimen to haploidentical regimens due to donor unavailability associated with COVID-19 pandemic. As a result, there were some haploidentical regimens with small sample size. On the other hand, these regimens provided possible superiority of PTCy to methotrexate to prevent OM.

In summary, the incidence of severe OM was largely different depending on the conditioning and immunosuppression regimens. Female gender was found to be a risk factor. In clinical trials including patients undergoing different HSCT regimens, it is best to stratify randomisation according to the regimens.

## Data Availability

All data and material are available on request.
